# W-SBA-15 as an Effective Catalyst for the Epoxidation of 1,5,9-Cyclododecatriene

**DOI:** 10.3390/molecules27248769

**Published:** 2022-12-10

**Authors:** Marcin Kujbida, Agnieszka Wróblewska, Grzegorz Lewandowski, Piotr Miądlicki, Beata Michalkiewicz

**Affiliations:** 1 Department of Catalytic and Sorbent Materials Engineering, Faculty of Chemical Technology and Engineering, West Pomeranian University of Technology in Szczecin, Piastów Ave. 42, 71-065 Szczecin, Poland; 2 Department of Chemical Organic Technology and Polymeric Materials, Faculty of Chemical Technology and Engineering, West Pomeranian University of Technology Szczecin, Piastów Ave. 42, 71-065 Szczecin, Poland

**Keywords:** 1,5,9-cyclododecatriene, 1,2-epoxy-5,9-cyclododecadiene, W-SBA-15, Ti-SBA-15, semi-bath process, epoxidation

## Abstract

The results of a study on the epoxidation of 1,5,9-cyclododecatriene (CDT) on a W-SBA-15 catalyst using the batch and half-periodic methods are presented. During this study, the activity of the W-SBA-15 catalyst was compared to that of the Ti-SBA-15 catalyst, and the W-SBA-15 catalyst was found to be about 20 times more active than the Ti-SBA-15 catalyst. The highest CDT conversion so far, amounting to 86 mol%, was obtained after carrying out the 4 h epoxidation process. Conducting the studied process using the semi-batch method did not result in the significant improvement in value functions describing this process (CDT conversion and selectivity of CDT transformation to ECDD), but the fastest H_2_O_2_ dosing rate (246 µL/h) allowed us to obtain 9 mol% higher CDT conversion in comparison to the batch method.

## 1. Introduction

Epoxy compounds are valuable raw materials for organic syntheses and the polymer industry. Examples of such compounds are ethylene oxide, propylene oxide, 1,2-epoxylimonene, and 1,2-epoxy-5,9-cyclododecadiene (ECDD). The last of these compounds, the obtaining of which was the aim of the research in this article, has found, inter alia, applications for the production of cyclododecanone, which is the raw material used for the production of laurolactam and dodecanedioic acid [1,2,3]. ECDD is currently finding newer applications, e.g., as a fragrance compound for the production of perfumes with the scent of musk, as well as a raw material for the production of other fragrances, e.g., 4,8-cyclododecadienone (the ingredient in perfumes with a woody–musky scent) [4,5]. 

ECDD can be obtained by the epoxidation processes using both homogeneous and heterogeneous catalysts. There are few literature reports on the use of heterogeneous catalysts in the epoxidation of 1,5,9-cyclododecatriene (CDT) to ECDD, although these catalysts have many advantages, e.g., an easy separation from post-reaction mixtures and a possibility of regeneration. Among the heterogeneous catalysts, the Ti-MCM-41 catalyst is used in the CDT epoxidation process. The main problem with the use of this catalyst is the low selectivity of the transformation of H_2_O_2_ to ECDD (the maximum achievable value amounts to 30 mol%) [6] and the relatively slow rate of CDT conversion in comparison to the processes which are conducted under phase transfer catalysis (PTC) conditions [1]. Further research on increasing the effective conversion of hydrogen peroxide (only part of the hydrogen peroxide is effectively converted to the epoxy compound; the rest undergoes ineffective decomposition under the conditions in which the reaction is carried out) may involve the use of a different mesoporous catalyst, e.g., one with the SBA-15 structure, and the introduction of a different metal into the catalyst structure, e.g., tungsten instead of titanium. An example of such a catalyst is the W-SBA-15 catalyst. It has been used so far in the following processes: photocatalytic decomposition of pollutants [7,8], CO_2_ reforming [9], hydrogenolysis [10], metathesis [11], and oxidation [12,13]. 

In our preliminary studies, which have not been published, we conducted the process of CDT epoxidation using titanium silicalite TS-1 as the catalyst. These studies showed that TS-1 is completely inactive in CDT epoxidation. This is most likely due to the small pore diameter of the TS-1 catalyst, which is smaller than the diameter of the CDT molecule. Thus, we decided to use in our studies a mesoporous catalyst with wider pores, with a very stable structure, and with tungsten incorporated into the silica–W-SBA-15 material. The aim of the research presented in this publication was to determine the activity of the W-SBA-15 catalyst in the CDT epoxidation process and to compare its activity to the activity of the Ti-SBA-15 catalyst. To the best of our knowledge, this work is the first report on the use of the mesoporous catalyst without using titanium atoms in the structure. We also present the results of experiments carried out using a semi-bath method performed by a dosing of H_2_O_2_ solution. In our opinion, both the use of the W-SBA-15 catalyst and the half-periodic method should result in a significant improvement in value functions describing the process of CDT conversion and selectivity of CDT transformation to ECDD. 

## 2. Results and Discussion 

### 2.1. Catalyst Characterization

**Figure 1:**
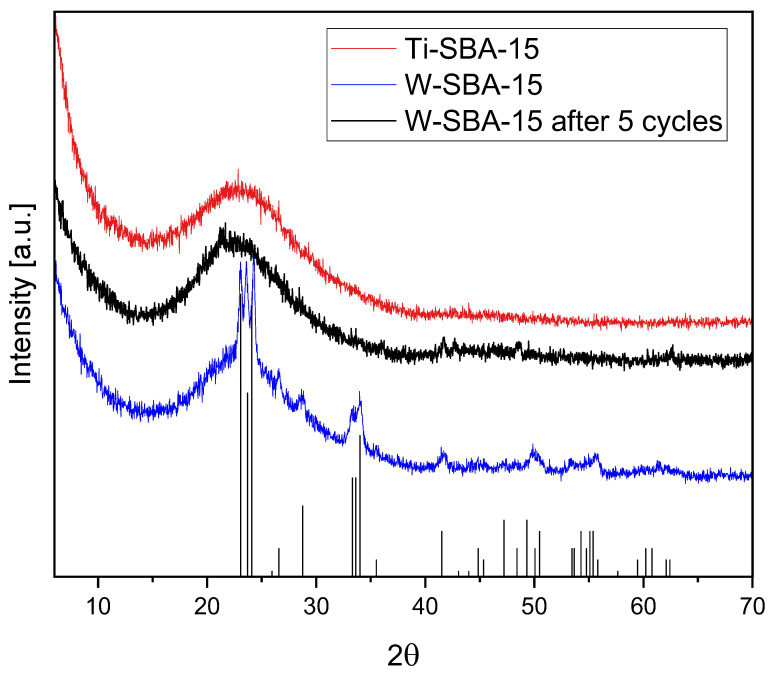
XRD patterns of Ti-SBA-15, W-SBA-15, and W-SBA-15 after 5 cycles. The black bars represent WO_3_ diffraction patterns according to the JPCDS card no. 20-1324.

The Ti-SBA-15 and W-SBA-15 catalysts were analyzed using the XRD method (Figure 1). A single, very broad peak was observed for both samples (Ti-SBA-15 and W-SBA-15) in the range of 2θ equal to 16–20°. Such a signal is characteristic of nearly amorphous silica [14], according to JCPDS card no. 82-1557. The characteristic peaks of an orthorhombic WO_3_ were detected in the patterns of W-SBA-15. It shows that not all the tungsten was built into the structure of the SBA-15 material. It was also clearly seen that after five cycles of catalytic tests, WO_3_ was completely removed. Simultaneously, no anatase and rutile diffraction patterns were present in the Ti-SBA-15 XRD spectra. Hence, the conclusion is that in the Ti-SBA-15 material, Ti was fully incorporated into the SBA-15 structure.

**Figure 2:**
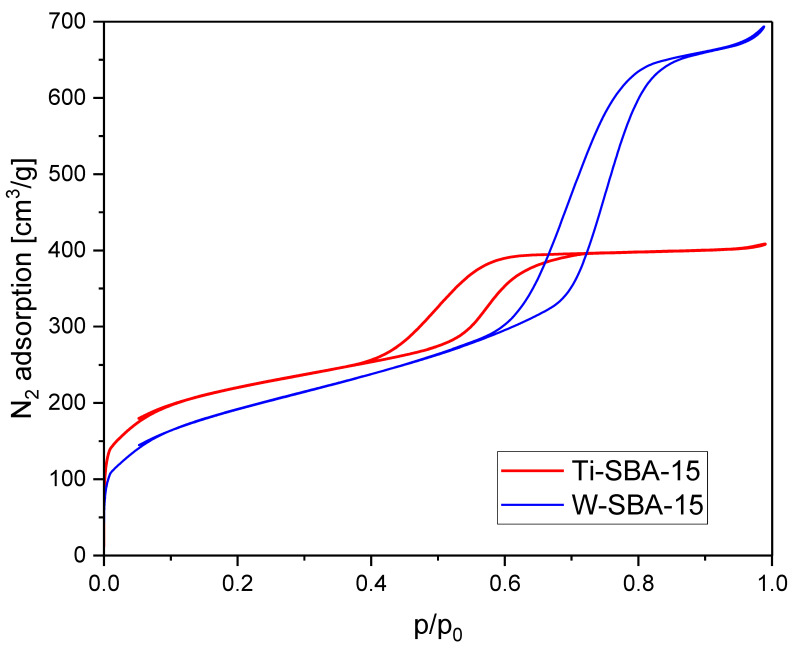
Nitrogen sorption isotherms of Ti-SBA-15 and W-SBA-15.

Nitrogen sorption isotherms of Ti-SBA-15 and W-SBA-15 are presented in Figure 2. Both isotherms displayed type IV with H1-type hysteresis loops typical of mesoporous materials with one-dimensional cylindrical pores [8]. The position of the hysteresis loop and the sharpness of the adsorption–desorption branches were different. The position where the hysteresis begins is relevant to pore sizes. The hysteresis loop of Ti-SBA-15 was placed in the range of p/p_0_ from 0.4 to 0.7. The hysteresis loop of W-SBA-15 was placed in the higher range of p/p_0_ from 0.57 to 0.87, and was distinctly sharper. The comparison of isotherm shapes indicated that W-SBA-15 exhibited much wider pores and a higher pore volume than Ti-SBA-15. 

**Figure 3:**
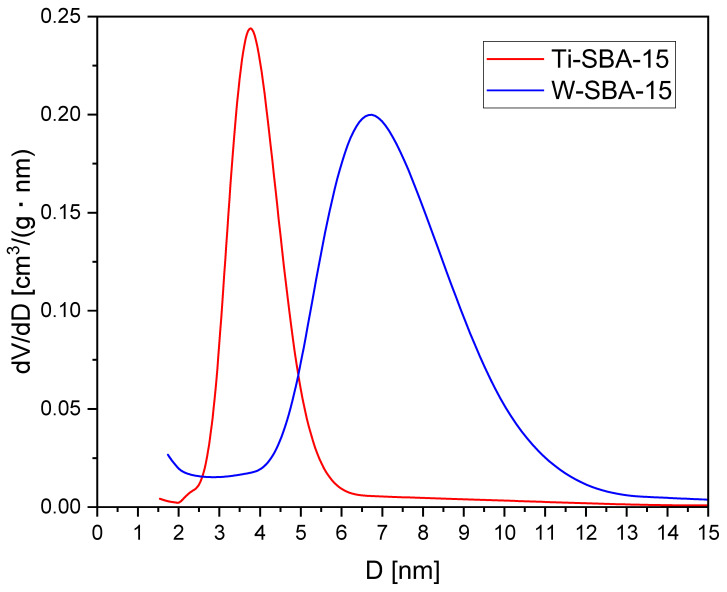
Pore size distribution calculated using the BJH method.

These conclusions are consistent with the BJH pore size distribution results presented in Figure 3 and the textural properties shown in Table 1. The pore size distribution for both samples was quite narrow: 2–6 nm for Ti-SBA-15 and 4–11 nm for W-SBA-15. The most common pore diameters found based on the maximum pore size distribution calculated using the BJH method were equal to 3.8 and 6.7 nm for the Ti-SBA-15 and W-SBA-15 catalysts, respectively. The widening of the pore size for W-SBA-15 is proof that part of the tungsten was incorporated into the SBA-15 structure. The W atomic radius is higher than Ti.

The specific surface area and micropore volume of W-SBA-15 were lower than those of Ti-SBA-15. The opposite behavior was observed for the total pore volume and pore diameters. Similar results were observed for W-SBA-15 and SBA-15 materials [15].

EDX analysis showed that Ti-SBA-15 contains 1.1 wt% Ti, and W-SBA-15 contains 1.8 wt% W. 

**Figure 4:**
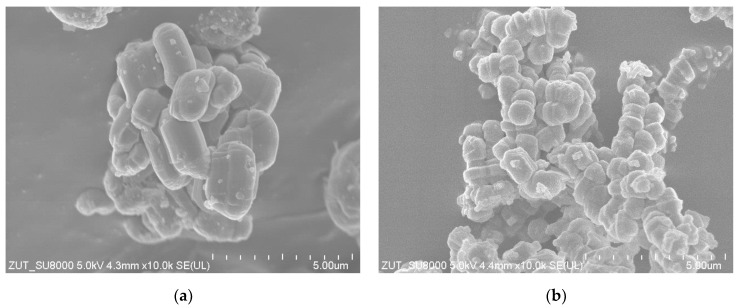
SEM images of Ti-SBA-15 (**a**) and W-SBA-15 (**b**).

The SEM image of Ti SBA-15 (Figure 4a) showed rod-shaped particles typical for the SBA-15 materials. The average diameter of Ti-SBA-15 particles was equal to 1 × 1.7 µm. The particles of W-SBA-15 (Figure 4b) were rather spherical, with an average diameter of about 1 µm. 

**Figure 5:**
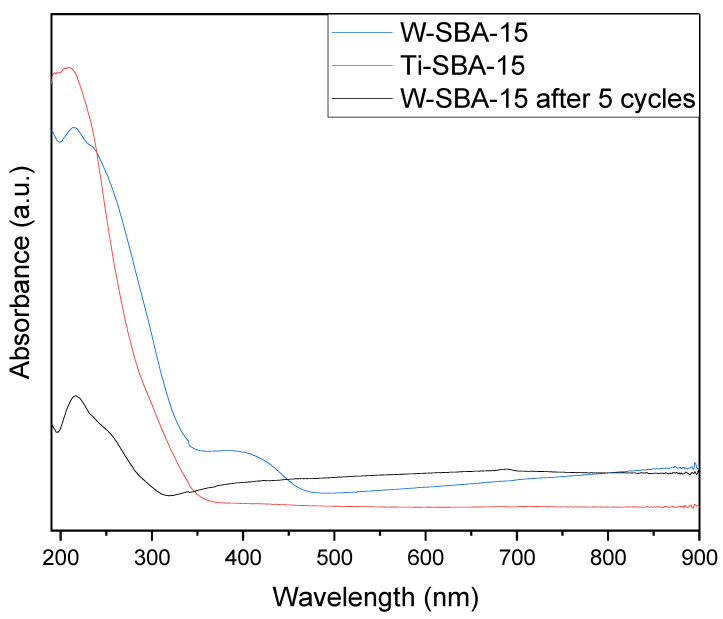
Diffuse reflectance UV-Vis spectra of the catalysts.

Figure 5 shows the Dr-UV-Vis spectra of the catalysts studied. In both cases, there was an absorbance maximum occurring around 220 nm associated with a ligand-to-metal charge transfer of isolated tetrahedral species, indicating effective incorporation of W and Ti atoms into the SBA-15 structure (this is proof of the presence of highly dispersed titanium and tungsten species in a silica framework). In the spectra of W-SBA-15, a low-intensity band at 250 nm (attributed to W^6+^) is also visible. This band indicates that partially polymerized W species with octahedral coordination and isolated W species with tetrahedral coordination coexisted in the support. In the W-SBA-15 materials spectrum, an additional peak with a maximum of around 420 nm is noticeable, probably associated with the presence of extra-framework WO_3_ clusters [16]. In the spectrum of the W-SBA-15 catalyst examined after five cycles, a decrease in the intensity of the 220 nm and 420 nm bands is clearly visible, which indicates the leaching of both W species incorporated into the SBA-15 structure ([WO_4_] tetrahedral species) and WO_3_ embedded in the catalyst pores. The UV-Vis results confirmed the XRD findings. 

**Figure 6:**
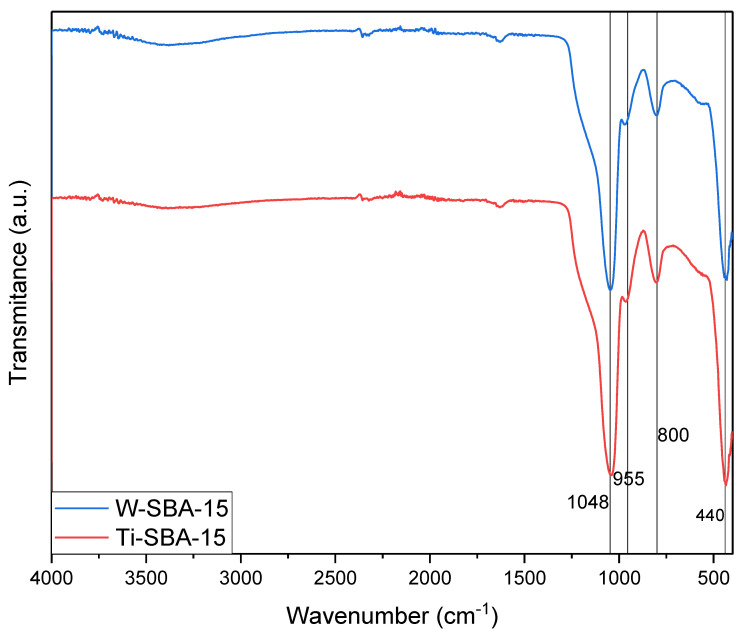
FT-IR spectra of the catalysts.

On the FT-IR spectra (Figure 6), all bands associated with the silica structure of SBA-15 are present (1048, 800, and 440 cm^−1^), as well as the band at 955 cm^−1^ (Si-O-W bond-stretching vibrations), which is an indicator of the effective incorporation of additional metal atoms into the structure of the material. There are no noticeable differences between the IR spectra of the two catalysts.

### 2.2. Catalytic Tests

**Figure 7:**
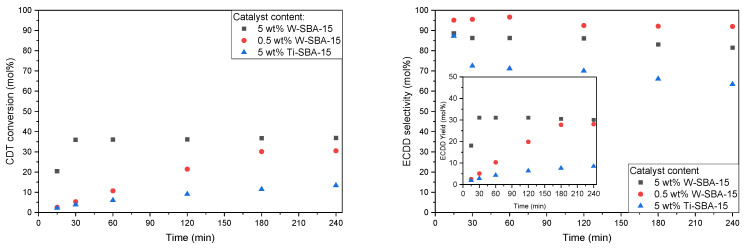
Graphs showing conversion of CDT (**left**), selectivity of transformation of CDT to ECDD (**right**, main), and ECDD yield in relation to CDT (**right**, insert) with different catalysts and catalyst contents. Reaction conditions: temperature, 60 °C; solvent, i-PrOH, 90 wt%; CDT:H_2_O_2_ (60 wt% aqueous solution) molar ratio, 2.

As can be seen on the CDT conversion graph (Figure 7), there was a significant difference in the activity of the two studied catalysts. W-SBA-15 showed significantly higher activity in the studied process in comparison to its titanium analog (Ti-SBA-15). The CDT conversion obtained for W-SBA-15 and Ti-SBA-15 was 36 and 13 mol%, respectively; however, for W-SBA-15, this value was obtained after 30 min and for Ti-SBA-15 only after 4 h. Thus, even when running the reaction with the 0.5 wt% W-SBA-15 catalyst, 2,3 times higher CDT conversion was obtained after the same reaction time.

Additionally, in the case of the selectivity of the transformation of CDT to ECDD (Figure 7), a significant advantage of the W-SBA-15 catalyst in comparison to the Ti-SBA-15 catalyst was visible. In the case of running the process with the 5 wt% catalyst, for the W-SBA-15 material, a roughly 15 mol% higher value of ECDD selectivity was obtained for almost the entire duration of the process (except for 15 min). The highest selectivity was obtained by conducting the process with the 0.5 wt% W-SBA-15 catalyst.

The molar ratio of CDT:H_2_O_2_ is a key parameter determining the final olefin conversion. It also determines the selectivity of the formation of epoxide compounds. With an excess of H_2_O_2_, the probability that more than one double bond in the CDT molecule will be oxidized increases. In this series of studies, we decided to test three molar ratios of CDT:H_2_O_2_: 2, 1, and 0.5. 

**Figure 8:**
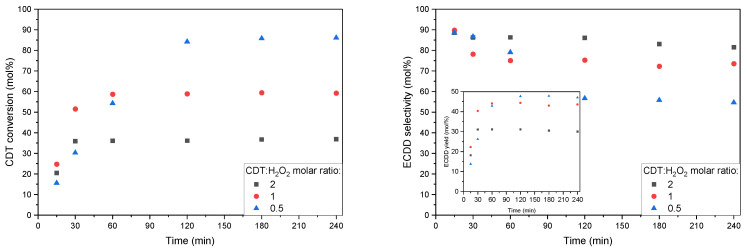
Graphs showing conversion of CDT (**left**), selectivity of transformation of CDT to ECDD (**right**, main), and ECDD yield in relation to CDT (**right**, insert) at different CDT:H_2_O_2_ molar ratios. Process conditions: temperature, 60 °C; solvent, i-PrOH, 90 wt%; H_2_O_2_ (60 wt% aqueous solution); catalyst content, 5 wt%.

Figure 8 shows the results of the aforementioned tests for the W-SBA-15 catalyst. An increase in the H_2_O_2_ content in the reaction mixture was seen to increase the conversion of CDT. However, these increases did not correspond to any of the 2-fold decreases in the CDT:H_2_O_2_ molar ratio. Changing the CDT:H_2_O_2_ molar ratio from 2 to 1 increased CDT conversion by 1.6 times, and from 1 to 0.5 by 1.4 times. This indicates an increasingly inefficient use of H_2_O_2_. 

The different processes conducted in this series of experiments differ in the time after which maximum CDT conversion is achieved. It is easy to see that a 2-fold decrease in the CDT:H_2_O_2_ molar ratio also results in a 2-fold increase in the time needed to reach maximum CDT conversion. It is interesting to note that the rate of increase in CDT conversion for the process carried out with the highest H_2_O_2_ content was the lowest. 

In the graph showing changes in the selectivity in obtaining ECDD, a downward trend is noticeable. It continued from the beginning of the process to the time corresponding to the obtaining of the maximum CDT conversion. Once the minimum is reached, selectivity stabilization occurs. Stopping the decrease in selectivity indicates that ECDD is largely resistant to epoxy ring hydration. The decrease in selectivity is associated with side reactions in which one of the reactants is H_2_O_2_.

**Figure 9:**
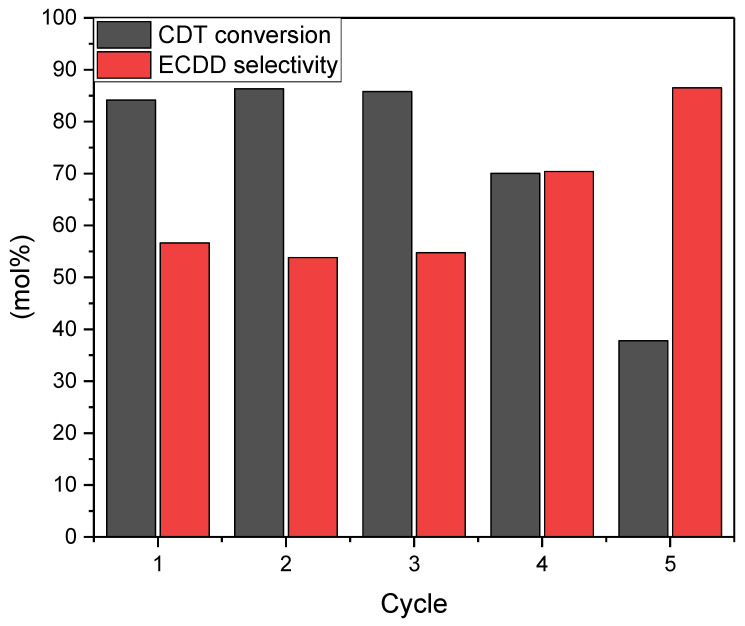
Graph showing changes in CDT conversion and selectivity of CDT to ECDD conversion as a function of the number of catalyst cycles. Process conditions: temperature, 60 °C; solvent, i-PrOH, 90 wt%; CDT:H_2_O_2_ molar ratio (60 wt% aqueous solution), 0.5; catalyst, W-SBA-15, 5 wt%.

The recycle test of the W-SBA-15 catalyst (Figure 9) showed that it had a stable activity for three cycles, after which there was a significant visible decrease in its activity. Because of the way the experiment was conducted, a decrease in activity due to the clogging of pores by polymers formed as by-products can be ruled out. The leaching of tungsten atoms from the catalyst structure, as occurs with many other catalysts of this type, is very probable [17]. This hypothesis may be confirmed by the DR-UV-Vis spectrum analysis carried out after the fifth catalyst cycle (Figure 5), which showed a significant reduction in signal intensity at 220 nm, indicating reduced W content in the sample.

It can be assumed that CDT, which has a much larger molecule than H_2_O_2_, will diffuse more slowly into the pores of the catalyst, so the epoxidation reaction will be limited mainly by the rate of its diffusion, and the diffusion of H_2_O_2_ will have a negligible effect on the reaction rate. In addition, there may likely be more H_2_O_2_ molecules than CDT molecules in the pores of the catalyst at the same time, which in turn may cause a decrease in selectivity by downstream reactions in which H_2_O_2_ is involved (as studies on the effect of CDT:H_2_O_2_ molar ratio showed). 

**Figure 10:**
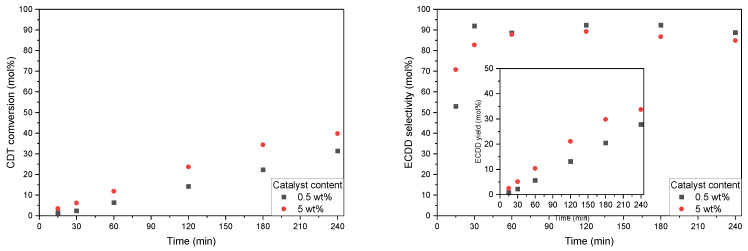
Graphs showing conversion of CDT (**left**), selectivity of transformation of CDT to ECDD (**right**, main), and ECDD yield in relation to CDT (**right**, insert) at different catalyst contents in the processes conducted using a semi-bath method. Process conditions: temperature, 60 °C; solvent, i-PrOH, 90 wt%; catalyst, W-SBA-15; CDT:H_2_O_2_ (9.54 wt% i-PrOH solution) molar ratio, 2.

Figure 10 shows the effect of the catalyst content at the constant H_2_O_2_ dosing rate on the conversion of CDT, the selectivity of its transformation to ECDD, and the yield of obtaining ECDD from CDT. A steep, linear increase in CDT conversion was observed with no accompanying decrease in selectivity. The initial low selectivity can be explained by measurement error due to the low CDT conversion (about 1 mol%) in the first 15 min of the process performing. The final results are very similar to those obtained for the processes carried out using the batch method. 

**Figure 11:**
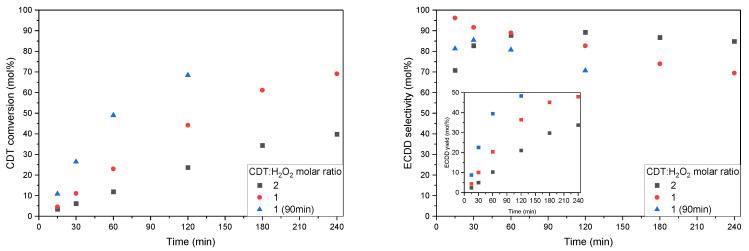
Graphs showing conversion of CDT (**left**), selectivity of transformation of CDT to ECDD (**right**, main), and ECDD yield in relation to CDT (**right**, insert) at different CDT:H_2_O_2_ molar ratios and H_2_O_2_ flow ratio in the processes conducted using a semi-bath method. Process conditions: temperature, 60 °C; solvent, i-PrOH, 90 wt%; catalyst, W-SBA-15, 5wt%; CDT:H_2_O_2_ (9.54 wt% i-PrOH solution) molar ratio, 2. H_2_O_2_ solution flow ratio: 2, 94 µL/h; 1, 105 µL/h; 1 (90 min), 246 µL/h.

Figure 11 shows the results of the studies on the effect of changing the CDT:H_2_O_2_ molar ratio and the H_2_O_2_ dosing rate. It can be seen that, similar to the batch conditions, the amount of H_2_O_2_ added to the reactor is crucial for the increase in the rate of CDT conversion as well as for the ECDD selectivity. 

A more than 2-fold increase in the H_2_O_2_ dosing rate also resulted in a similarly rapid increase in the rate of CDT conversion gain, resulting in the same final results after 2 h of running the process as after 4 h of slower dosing. Faster dosing yielded 9 mol% higher CDT conversion compared to the batch method with only 3 mol% lower ECDD selectivity. This indicates the more efficient use of H_2_O_2_ with the half-periodic method. 

## 3. Materials and Methods

### 3.1. Catalyst Synthesis

The W-SBA-15 catalyst with the molar ratio of Si:W = 30:1 was obtained using the method described by Chang et al. [8]. According to this method, 5.51 g of Pluronic 123 (Average Mn ~5800, Aldrich, St. Louis, MO, USA) was dissolved in 179 cm^3^ of 2 M aqueous solution of HCl. The mixture was then stirred overnight at 35 °C, after which 11.852 g of tetraethyl orthosilicate (98%, Aldrich, Poznań, Poland) was added to it. After 30 min, 9 cm^3^ of 0.2 M aqueous solution of Na_2_WO_4_*2H_2_O (98%, Angene, Nanjing, China) was added to the reactor. After 24 h of stirring at 35 °C, the mixture was transferred to a Teflon-lined autoclave and kept at 100 °C for 48 h. The contents of the autoclave were then drained under reduced pressure and washed with deionized water and methanol. The resulting material was dried at 60 °C for 24 h and then calcined at 550 °C for 5 h.

The Ti-SBA-15 catalyst with the molar ratio of Si:Ti = 30:1 in the crystallization gel used for comparison of the activity between W-SBA-15 and Ti-SBA-15 materials was obtained according to the method described by Berube et al. [18].

### 3.2. Catalyst Characterization

The X-ray diffraction (XRD) patterns were recorded on X’Pert–PRO, Panalytical, Almelo, The Netherlands, 2012 using Cu Kα radiation.

The N_2_ sorption isotherms were measured at −196 °C using an ASAP Sorption Surface Area and Pore Size Analyzer (ASAP 2460, Micrometrics, Norcross, GA, USA 2018).

Scanning electron microscopy was performed with an SU8020 Ultra-High Resolution Field Emission Scanning Electron Microscope (Hitachi Ltd., Ibaraki, Japan, 2012). The elemental analysis was conducted using Energy-Dispersive X-ray spectrometers (EDX) with the same instrument. The samples for SEM were sputter-coated with 40 nm of chromium in order to reduce charging.

FT-IR spectra of the catalyst were obtained using a Thermo Finnigan Nicolet 380 FT-IR instrument with an ATR Smart iTX attachment (Thermo Fisher Scientific Inc., Waltham, MA, USA) in the wavenumber range from 490 to 4000 cm^−1^. 

Diffuse reflectance UV-Vis spectra of the catalyst in the wavelength range from 190 to 900 nm were obtained using a Jasco 650 (V-650, Jasco, Tokyo, Japan) spectrometer with a PIV-756 horizontal integrating sphere. 

### 3.3. Catalytic Tests

All epoxidations performed using the batch method were conducted in a 5 cm^3^ glass reactor with a magnetic stirrer and equipped with a reflux condenser. The reactor was placed in an oil bath at the desired temperature. In a typical synthesis, the following reagents were successively added to the reactor: 60 wt% water solution of hydrogen peroxide (analytical grade, Chempur, Piekary Śląskie, Poland), followed by isopropanol (i-PrOH)–solvent (analytical grade, Stanlab, Lublin, Poland), c,t,t-1,5,9-cyclododecatriene (98%, Aldrich, Poznań, Poland), dodecane(99+%, Alfa Aesar, Kandel, Germany, internal standard), and the catalyst. 

Epoxidations carried out using the half-periodic method were performed in a glass reactor (a three-necked heart-shaped flask) with a 25 cm^3^ capacity, equipped with the magnetic stirrer and the reflux condenser, and placed in the oil bath at a preset temperature. During the typical synthesis, appropriate amounts of CDT, dodecane, and i-PrOH were added sequentially to the reactor. A 9.54 wt% solution of H_2_O_2_ in i-PrOH (prepared by dissolving the appropriate amount of 60 wt% H_2_O_2_) was dosed into the reactor using a syringe pump with a volumetric flow to ensure equal dosing of the solution over 3.5 h.

The catalyst recycle tests were carried out as follows. After the reaction, the catalyst was separated by centrifugation from the liquid, and then shaken with about 5 cm^3^ of i-PrOH. The steps were repeated 3 times; then, the catalyst was dried at 100 °C and calcined at 550 °C for 5 h.

The samples of the reaction mixture were taken at the beginning of the process (before adding the catalyst), and after 15, 30, 60, 180, and 240 min, and centrifuged and diluted with acetone at a volume ratio of 1:10, followed by chromatographic analysis.

After the process, the reactor contents were centrifuged, and the hydrogen peroxide conversion was determined using the iodometric method.

## 4. Conclusions

The study clearly indicates that the SBA-15-type catalyst having a tungsten atom as the active center is much more active in CDT epoxidation than its titanium counterpart.

The substitution of Ti for W in the catalyst structure results in the increase in pore size, most likely due to the larger size of the W atom. The higher activity of W-SBA-15 may not only be due to the higher activity of W itself but may also be due to the 1.8-fold-larger pore diameter facilitating internal mass transport.

By running the studied process on the W-SBA-15 catalyst, it was possible to obtain a reaction rate not previously reported—36 mol% of CDT conversion after 30 min of running the process with the 5 wt% catalyst. In tests conducted with different molar ratios of CDT:H_2_O_2_, it was possible to obtain the highest CDT conversion to date for the process carried out using heterogeneous catalysis. It was equal to 86 mol%. Conducting the investigated process using the half-period method did not result in a significant improvement in the process results. However, by dosing the H_2_O_2_ solution at the rate of 246 µL/h, it was possible to obtain a 9 mol% higher CDT conversion with a selectivity comparable to that obtained by the batch method.

The catalyst recycle test showed that it has stable activity for three cycles, followed by a gradual decrease in activity due to the leaching of W atoms from the catalyst structure. This was confirmed by the DR-UV-Vis analysis of the recycled catalyst.

The presented results, in our opinion, represent a significant advance in the study of CDT epoxidation on heterogeneous catalysts and should be continued. Further research should primarily focus on increasing the durability of the catalyst and increasing the number of reaction cycles in which the catalyst is active.

## Figures and Tables

**Table 1:** Textural parameters of Ti-SBA-15 and W-SBA-15.

Catalyst	S_BET_ [m^2^/g]	V_tot_ [cm^3^/g]	V_mic_ [cm^3^/g]:
Ti-SBA-15	773	0.634	0.165
W-SBA-15	664	1.08	0.052

## Data Availability

Experimental data are available from the authors.

## Sample Availability

Samples of the compounds Ti-SBA-15 and W-SBA-15 are available from the authors.
